# Beastly Beauty Products: Exposure to Inorganic Mercury in Skin-Lightening Creams

**DOI:** 10.1289/ehp.119-a80b

**Published:** 2011-02

**Authors:** Cynthia Washam

**Affiliations:** **Cynthia Washam** writes for *EHP*, *Oncology Times*, and other science and medical publications from South Florida

The most common exposure to mercury is from organic methylmercury found in fish. People with no workplace exposure to mercury typically have low levels of exposure to inorganic or elemental mercury, although nonoccupational exposure can occur from dental amalgams, some herbal medicine products, and cosmetics that contain mercury. When a New York City (NYC) biomonitoring study revealed that thousands of women in that city may have been exposed to dangerous levels of inorganic mercury from imported skin-lightening creams, city health officials enlisted the help of U.S. and international health agencies in getting the creams off local store shelves [***EHP*** **119(2):203–209; McKelvey et al.]**.

In 2004 the NYC Department of Health and Mental Hygiene (DOHMH) conducted the nation’s first local Health and Nutrition Examination Survey. Analysis of urine specimens from 1,840 adult New Yorkers collected during the survey yielded a geometric mean mercury concentration of 0.73 μg/L, slightly higher than the national average of 0.5 μg/L. The authors took note when 13 women were found to have urine mercury concentrations exceeding the state’s reportable level of 20 μg/L; 4 women had levels exceeding 50 μg/L.

All 13 highly exposed women were Hispanic or black, and 10 had been born in the Dominican Republic. Each of the 9 women interviewed on followup had used mercury-containing skin-lightening cream. One such product sampled by DOHMH workers contained 6,190 ppm mercury. The U.S. Food and Drug Administration (FDA) limit for mercury in skin-care products is 1 ppm.

Extrapolating from the population sampled, the authors estimate nearly 27,000 New Yorkers may have urine mercury levels exceeding 20 μg/L. Although the researchers did not assess potential health effects among the highly exposed women, occupational studies indicate kidney and neurologic toxicity may occur when urine mercury levels exceed 20 μg/L.

City health officials responded to the survey results by seizing 12 brands of illegally imported cosmetics from store shelves. All the products listed mercury as an active ingredient. Press releases issued by the DOHMH urged residents to report mercury-tainted cosmetics and the shops selling them, and New Jersey investigators were enlisted to plug the pipeline to importers in that state. The Pan American Health Organization called on the Dominican Republic to stop manufacturing the dangerous products. The Dominican Secretary of Health reportedly has notified all laboratories to stop manufacturing mercury-containing skin-care products.

The authors realize some tainted products may still cross the border, as they have for years despite FDA prohibitions. But they believe their efforts, coupled with evidence of mercury’s toxicity and continued vigilance, will substantially reduce the availability and use of these products.

## Figures and Tables

**Figure:**
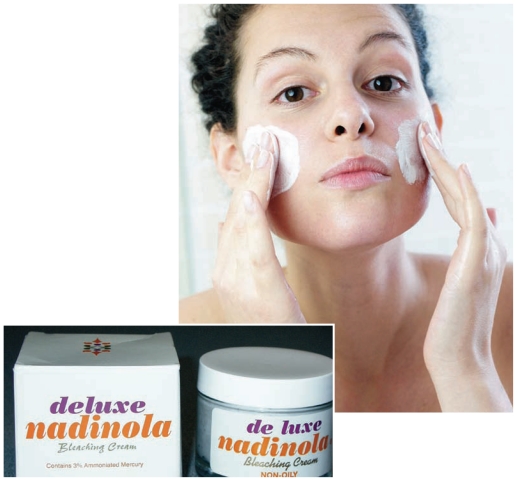
Only half the sampled products listed mercury on the label. Most exceeded the FDA limit for mercury in personal care products by several hundred or thousand times.

